# Supplementary figures and images for: Sensorimotor vs. Motor Upper Limb Therapy for Patients With Motor and Somatosensory Deficits: A Randomized Controlled Trial in the Early Rehabilitation Phase After Stroke
Source: Front Neurol. 2020 Dec 4;11:597666. doi: 10.3389/fneur.2020.597666 (PMC7746814; doi:10.3389/fneur.2020.597666)

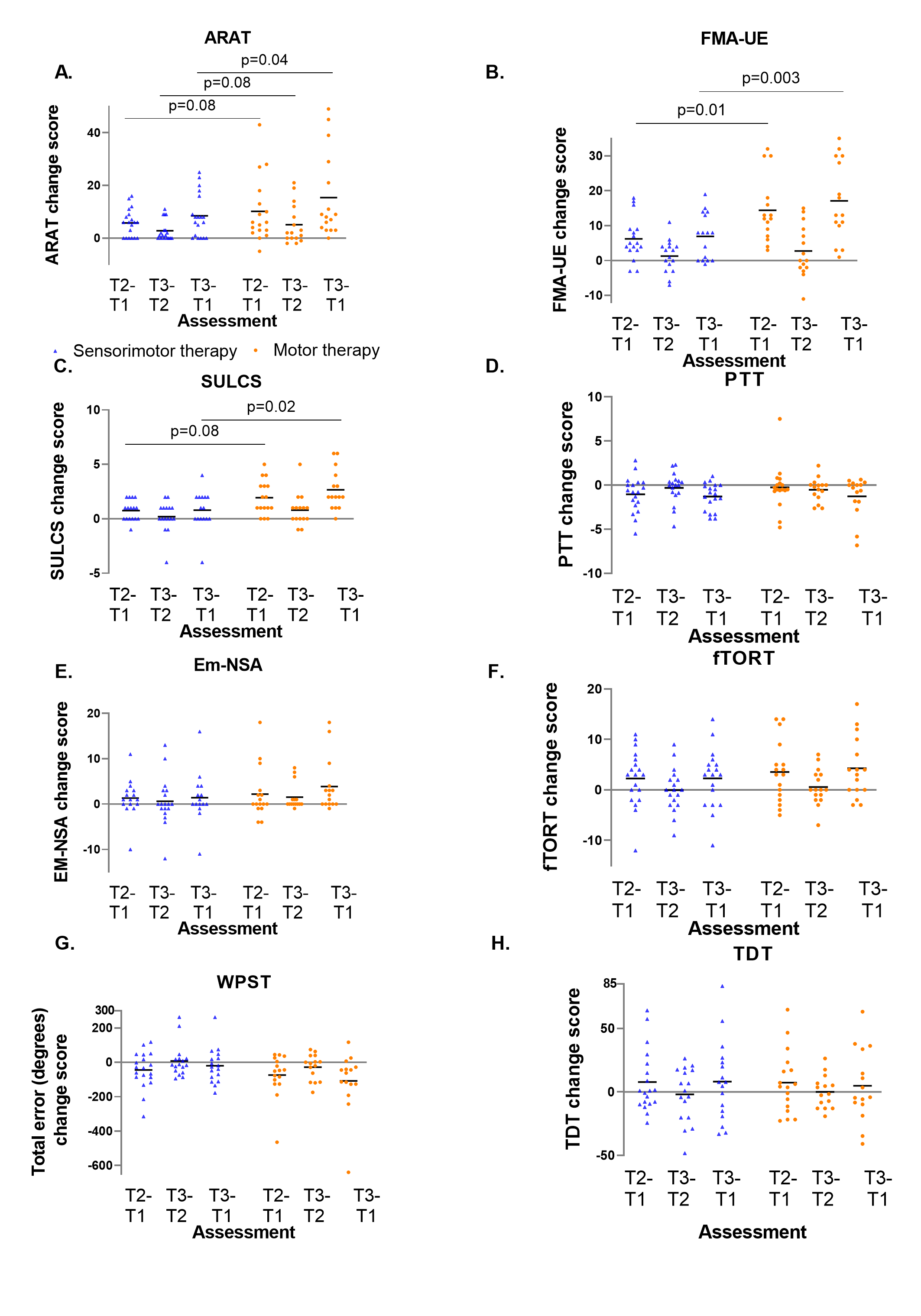

Supplement: Supplementary Figure 1 — Individual delta changes over time; every dot (motor therapy) or triangle (sensorimotor therapy) at one time point represents the delta change score of a patient; raw median scores indicated with horizontal bar. (A) ARAT: Action Research Arm Test, (B) FMA-UE: Fugl-Meyer assessment upper extremity part, (C) SULCS stroke upper limb capacity scale, (D) PTT: perceptual threshold of touch (mA), (E) Em-NSA: Erasmus modification of Nottingham Sensory Assessment, (F) fTORT functional tactile object recognition test, (G) WPST: wrist position sense test mean error (degrees), (H) TDT_AUC: texture discrimination test area under curve score. [file Image_1.TIF]

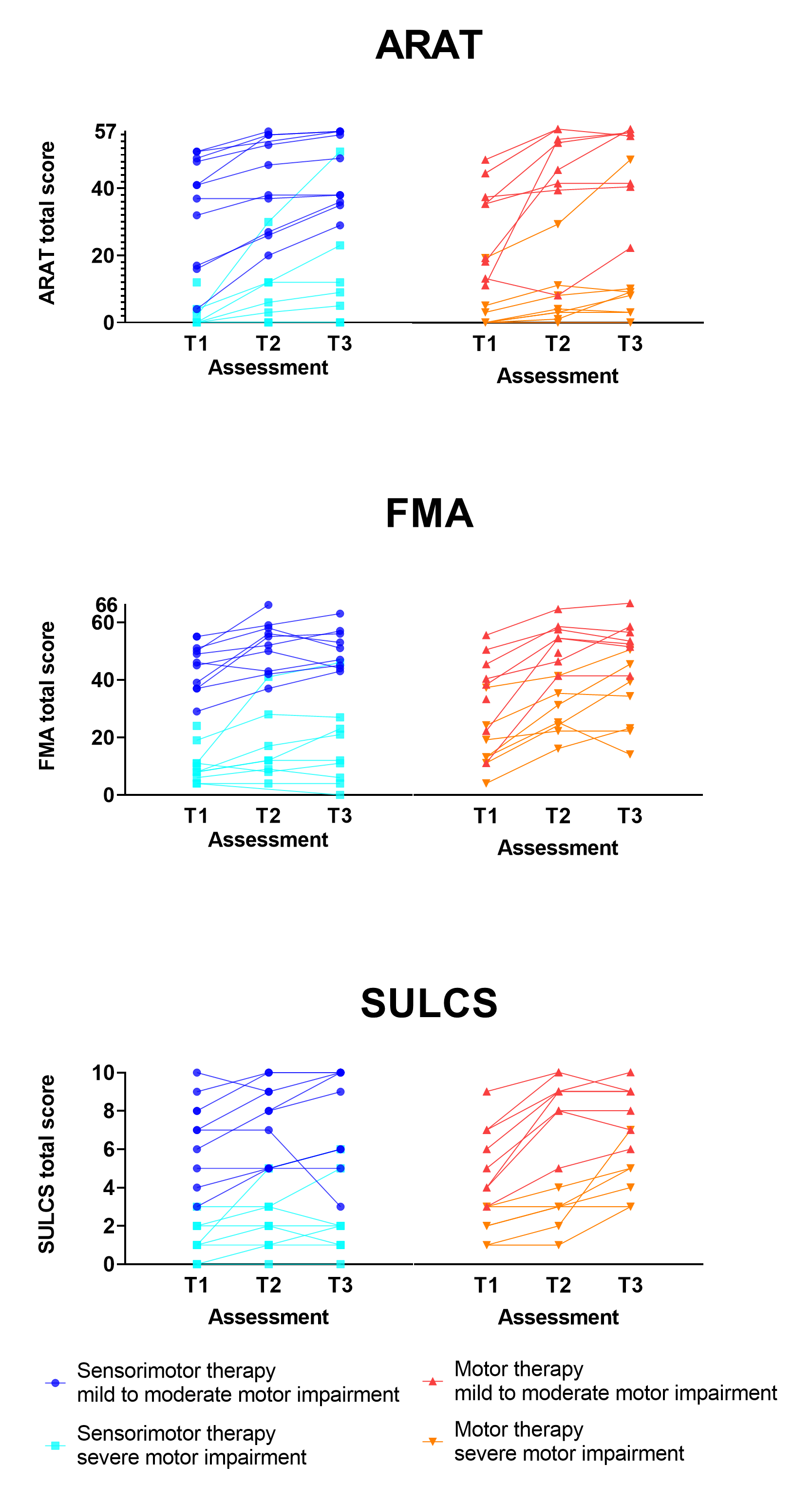

Supplement: Supplementary Figure 2 — Individual time courses of motor recovery. Every dot/square (sensorimotor therapy) or triangle (motor therapy) at one time point represents the raw value of a patient. Subdivision is made for patients with mild to moderate and severe baseline motor impairments. ARAT, Action Research Arm Test; FMA-UE, Fugl-Meyer assessment upper extremity part; SULCS, stroke upper limb capacity scale. [file Image_2.tif]
