# Supplementary material for: Sensorimotor vs. Motor Upper Limb Therapy for Patients With Motor and Somatosensory Deficits: A Randomized Controlled Trial in the Early Rehabilitation Phase After Stroke
Source: Front Neurol. 2020 Dec 4;11:597666. doi: 10.3389/fneur.2020.597666 (PMC7746814; doi:10.3389/fneur.2020.597666)
Supplement: Supplementary Table 1 — Subgroup analysis of baseline motor impairments of between group comparisons of an intervention effect corrected for age. [file Table_1.pdf]

Supplementary table 1. Subgroup analysis based on baseline motor impairments of between group comparison of an intervention effect corrected for age

MILD TO MODERATE baseline motor impairment

Motor function

		**ARAT** /57	**FMA** /66	**SULCS** /10	**ABILDHAND** (logits)
**T2-T1**	Sensorimotor group	7.52 (3.53)	7.83 (2.78)	1.24 (0.37)	5.32 (2.25)
	Motor group	14.64 (3.92)	16.34 (3.32)	2.71 (0.39)	6.48 (2.35)
	p-value	0.20	0.07	**0.01***	0.73
	95%CI	(-18.42-4.17)	(-17.86-0.86)	(-2.62--0.34)	(-8.51 -6.19)
	Effect size (G_{Hedges})	-0.64	-0.97	-1.33	-0.17
**T3-T2**	Sensorimotor group	3.36 (1.74)	0.64 (1.82)	0.43 (0.34)	5.77 (2.62)
	Motor group	4.92 (2.04)	0.62 (2.10)	-0.19 (0.38)	2.78 (2.99)
	p-value	0.59	1.00	0.25	0.48
	95%CI	(-7.58-4.46)	(-6.13-6.17)	(-0.43-1.65)	(-5.71 -11.68)
	Effect size (G_{Hedges})	-0.29	0.003	0.61	0.38
**T3-T1**	Sensorimotor group	11.13 (3.76)	7.76 (2.88)	1.37(0.96)	11.42 (2.54)
	Motor group	19.82 (4.52)	16.77 (3.29)	2.69 (1.05)	8.12 (2.77)
	p-value	0.16	0.06	0.36	0.41
	95%CI	(-21.24-3.85)	(-18.45-0.43)	(-4.12-1.48)	(-4.97 -11.57)
	Effect size (G_{Hedges})	-0.73	-1.04	-0.48	0.45

Somatosensory function

		**Em-NSA** /40	**PTT** /10mA	**TDT**-AUC	**WPST** total error degrees	**WPST** mean error degrees	**fTORT /42**
**T2-T1**	Sensorimotor group	3.46 (1.53)	-1.97 (0.83)	18.01 (8.25)	-77.98 (29.05)	-2.18 (1.06)	2.98 (1.80)
	Motor group	0.54 (1.53)	0.37 (0.92)	10.18 (9.09)	-50.43 (33.64)	-3.18 (1.21)	2.90 (1.99)
	p-value	0.22	0.08	0.54	0.55	0.55	0.98
	95%CI	(-1.93-7.77)	(-5.00-0.32)	(-19.06-37.73)	(-123.67-68.57)	(-2.47-4.47)	(-5.71-5.86)
	Effect size (G_{Hedges})	0.67	-0.90	0.30	-0.29	0.31	0.01
**T3-T2**	Sensorimotor group	0.28 (1.53)	0.53 (0.42)	3.85 (5.99)	-6.08 (24.51)	0.18 (1.15)	-0.35 (0.98)
	Motor group	1.52 (1.76)	-0.33 (0.53)	-6.17 (7.43)	28.50 (28.34)	1.01 (1.30)	-0.29 (1.14)
	p-value	0.62	0.25	0.34	0.39	0.65	0.97
	95%CI	(-6.41-3.93)	(-0.69-2.40)	(-11.60-31.65)	(-118.43-49.27)	(-4.58-2.92)	(-3.45-3.33)
	Effect size (G_{Hedges})	-0.27	0.67	0.55	-0.47	-0.24	-0.02
**T3-T1**	Sensorimotor group	1.10 (1.86)	-1.27 (0.62)	18.28 (9.67)	-30.86 (27.35)	-2.18 (1.06)	2.10 (2.10)
	Motor group	2.79 (1.93)	-1.23 (0.82)	4.03 (11.96)	-53.57 (31.62)	-3.18 (1.21)	3.57 (2.39)
	p-value	0.55	0.97	0.38	0.61	0.55	0.65
	95%CI	(-7.52-4.13)	(-2.28-2.29)	(-19.00-47.50)	(-68.37-113.78)	(-2.47-4.47)	(-8.27-5.32)
	Effect size (G_{Hedges})	-0.34	-0.02	0.49	-1.02	0.31	-0.23

SEVERE baseline motor impairment

**Motor function**		**ARAT** /57	**FMA** /66	**SULCS** /10	**ABILDHAND** (logits)
**T2-T1**	Sensorimotor group	1.84 (2.55)	2.90 (3.21)	0.06 (0.44)	5.57 (0.92)
	Motor group	8.05 (2.55)	13.59 (2.96)	1.32 (0.41)	0.45 (0.94)
	p-value	0.13	**0.05**	0.09	**0.01***
	95%CI	(-14.56-2.12)	(-21.26--0.11)	(-2.72-0.20)	(1.64 -8.61)
	Effect size (G_{Hedges})	-0.81	-1.27	-1.08	1.40
**T3-T2**	Sensorimotor group	0.39 (2.40)	1.19 (3.24)	-0.22 (0.61)	5.29 (1.61)
	Motor group	7.17 (2.4)	5.46 (2.99)	1.82 (0.57)	-3.43 (1.70)
	p-value	0.09	0.40	**0.04**	**0.002***
	95%CI	(-14.73-1.18)	(-14.79-6.24)	(-3.93--0.15)	(3.53-13.92)
	Effect size (G_{Hedges})	-0.94	-0.50	-1.26	0.46
**T3-T1**	Sensorimotor group	3.61 (3.99)	4.90 (4.03)	0.21 (0.58)	8.17 (1.64)
	Motor group	13.83 (3.99)	18.34 (3.73)	2.82 (0.54)	-0.56 (1.67)
	p-value	0.10	**0.03**	**0.01***	**0.002***
	95%CI	(-22.49-2.05)	(-25.72--1.14)	(-4.38--0.85)	(3.57-13.89)
	Effect size (G_{Hedges})	-0.85	-1.27	-1.70	0.86

Somatosensory function

		**Em-NSA** /40	**PTT** /10mA	**TDT**-AUC	**WPST** total error degrees	**WPST** mean error degrees	**fTORT /42**
**T2-T1**	Sensorimotor group	-0.29 (2.22)	-0.26 (0.66)	-3.34 (9.39)	-37.06 (57.09)	-1.56 (2.63)	1.79 (2.33)
	Motor group	3.61 (2.23)	-0.61 (0.66)	5.67 (9.32)	-67.69 (57.09)	-3.67 (2.63)	3.99 (2.33)
	p-value	0.26	0.72	0.52	0.73	0.59	0.53
	95%CI	(-10.96-3.17)	(-1.73-2.44)	(-37.73-19.71)	(-151.21-212.47)	(-6.07-10.30)	(-9.35-4.95)
	Effect size (G_{Hedges})	-0.62	0.18	-0.32	0.19	0.28	-0.31
**T3-T2**	Sensorimotor group	1.55 (1.73)	-1.37 (0.60)	-9.04 (5.88)	-15.06 (40.84)	-.47 (1.95)	0.35 (1.62)
	Motor group	1.23 (1.83)	-0.34 (0.60)	5.20 (5.55)	-43.19 (40.84)	-2.45 (1.95)	0.99 (1.62)
	p-value	0.91	0.27	0.12	0.67	0.51	0.80
	95%CI	(-5.43-6.06)	(-2.90-0.85)	(-32.71-4.25)	(-109.60-165.86)	(-4.30-8.27)	(-5.78-4.49)
	Effect size (G_{Hedges})	0.06	-0.58	-0.86	0.24	0.36	-0.13
**T3-T1**	Sensorimotor group	2.34 (2.26)	-1.52 (0.67)	-3.87 (9.59)	-40.06 (62.63)	-1.56 (2.63)	2.57 (2.10)
	Motor group	4.48 (2.27)	-1.05 (0.67)	6.18 (9.04)	-122.94 (62.63)	-3.67 (2.63)	4.54 (2.10)
	p-value	0.53	0.64	0.47	0.38	0.59	0.53
	95%CI	(-9.22-4.93)	(-2.54-1.59)	(-38.62-18.54)	(-111.37-277.13)	(-6.07-10.30)	(-8.42-4.46)
	Effect size (G_{Hedges})	-0.33	-0.23	-0.37	0.47	0.28	-0.31

Estimated mean and standard error of changes scores (T2-T1, T3-T2, T3-T1) are presented for both groups; p-values based on mixed models with age stroke onset (years) as covariate to evaluate differences between the change scores of both groups. Correction for multiple comparison was set on $p<0,02$. ARAT: action research arm test, FMA-UE: Fugl- Meyer assessment upper extremity section, SULCS: stroke upper limb capacity scale, Em-NSA: Erasmus modification of Nottingham sensory assessment, PTT: perceptual threshold of touch, TDT: texture discrimination test, AUC: area under curve, WPST: wrist position sense test, fTORT: functional tactile object recognition test.
