# Supplementary material for: Sensorimotor vs. Motor Upper Limb Therapy for Patients With Motor and Somatosensory Deficits: A Randomized Controlled Trial in the Early Rehabilitation Phase After Stroke
Source: Front Neurol. 2020 Dec 4;11:597666. doi: 10.3389/fneur.2020.597666 (PMC7746814; doi:10.3389/fneur.2020.597666)
Supplement: Supplementary Table 2 — Subgroup analysis of baseline texture discrimination impairments of between group comparisons of an intervention effect corrected for age. [file Table_2.pdf]

Supplementary table 2. Subgroup analysis based on baseline texture discrimination impairments of between group comparison of an intervention effect corrected for age

**MILD TO MODERATE baseline texture discrimination impairment**					
**Motor function**					
		**ARAT** /57	**FMA** /66	**SULCS** /10	**ABILDHAND** (logits)
**T2-T1**	Sensorimotor group	2.88 (4.27)	7.09 (3.22)	0.74 (0.74)	0.14 (2.14)
	Motor group	19.26 (4.27)	17.47 (3.46)	2.14 (0.68)	8.59 (1.96)
	p-value	**0.02**	0.05	0.21	**0.02**
	95%CI	(-29.50 --3.26)	(-20.91-0.14)	(-3.66-0.87)	(-15.02--1.87)
	Effect size (G_{Hedges})	-1.36	-1.14	-0.71	-1.51
**T3-T2**	Sensorimotor group	-1.25 (1.86)	-0.91 (2.32)	-0.16 (0.47)	-2.91 (1.93)
	Motor group	9.36 (2.00)	6.76 (2.51)	0.73 (0.52)	4.62 (2.12)
	p-value	**0.005**	0.06	0.27	**0.02**
	95%CI	(-17.31--3.91)	(-15.55-0.020)	(-2.56-0.78)	(-13.96--1.10)
	Effect size (G_{Hedges})	-2.01	-1.16	-0.66	-1.36
**T3-T1**	Sensorimotor group	4.75 (4.77)	5.96 (3.19)	0.17 (0.74)	0.42 (1.99)
	Motor group	26.57 (5.10)	24.47 (3.43)	3.01 (0.74)	10.36 (1.98)
	p-value	**0.007**	**0.002**	**0.020**	**0.004**
	95%CI	(-36.89 --6.73)	(-28.84--8.18)	(-5.17--0.50)	(-16.34--3.52)
	Effect size (G_{Hedges})	-1.62	-2.05	-1.45	-1.89

Somatosensory function

		**Em-NSA** /40	**PTT** /10mA	**TDT**-AUC	**WPST** total error degrees	**WPST** mean error degrees	**fTORT /42**
**T2-T1**	Sensorimotor group	0.49 (1.00)	-1.00 (0.43)	-13.01 (7.59)	-13 (31)	-0.29 (1.40)	4.86 (1.91)
	Motor group	0.33 (0.92)	-0.44 (0.43)	9.21 (7.40)	-24 (31)	-1.94 (1.50)	3.16 (1.90)
	p-value	0.92	0.39	0.08	0.82	0.46	0.56
	95%CI	(-3.21-3.54)	(-1.93-0.81)	(-47.34-2.90)	(-91 -112)	(-2.93-6.23)	(-4.37-7.77)
	Effect size (G_{Hedges})	0.06	-0.46	-1.05	0.13	0.42	0.32
**T3-T2**	Sensorimotor group	-1.29 (1.49)	0.31 (0.41)	-13.52 (9.25)	-35 (30)	-1.37 (1.45)	-1.02 (1.56)
	Motor group	1.78 (1.59)	-0.21 (0.44)	6.80 (9.18)	-25 (32)	-1.80 (1.56)	-0.61 (1.69)
	p-value	0.20	0.43	0.18	0.84	0.85	0.87
	95%CI	(-7.89 -1.76)	(-0.84-1.87)	(-50.26-9.62)	(-112-91)	(-4.29-5.16)	(-5.75-4.94)
	Effect size (G_{Hedges})	-0.73	0.45	-0.83	-0.12	0.1	-0.09
**T3-T1**	Sensorimotor group	-0.47 (1.48)	-0.86 (0.45)	-16.90 (9.25)	-23 (37)	-0.29 (1.40)	3.36 (2.12)
	Motor group	1.67 (1.50)	-0.47 (0.48)	2.78 (9.25)	-85 (40)	-1.94 (1.50)	3.42 (2.28)
	p-value	0.35	0.58	0.18	0.29	0.46	0.99
	95%CI	(-6.81 -2.53)	(-1.86-1.08)	(-49.23-9.87)	(-58-182)	(-2.93-6.23)	(-6.95-6.82)
	Effect size (G_{Hedges})	-0.54	-0.31	-0.80	0.59	0.42	-0.01

SEVERE baseline texture discrimination impairment

Motor function

		**ARAT** /57	**FMA** /66	**SULCS** /10	**ABILDHAND** (logits)
**T2-T1**	Sensorimotor group	6.24 (1.84)	4.91 (2.31)	1.01 (0.34)	6.43 (1.43)
	Motor group	4.13 (2.01)	12.12 (2.42)	1.49 (0.35)	2.59 (1.42)
	p-value	0.47	0.05	0.37	0.09
	95%CI	(-3.97-8.17)	(-14.55-0.13)	(-1.62 -0.66)	(-0.72-8.39)
	Effect size (G_{Hedges})	0.35	-1.05	-0.48	0.90
**T3-T2**	Sensorimotor group	4.09 (1.95)	3.36 (1.87)	0.85 (0.56)	9.18 (1.96)
	Motor group	3.80 (2.12)	-0.38 (1.89)	0.62 (0.56)	0.37 (1.97)
	p-value	0.92	0.20	0.78	**0.01**
	95%CI	(-6.01- 6.60)	(-2.19-9.66)	(-1.49-1.95)	(2.61-15.01)
	Effect size (G_{Hedges})	0.05	0.7	0.15	1.49
**T3-T1**	Sensorimotor group	9.49 (2.45)	7.64 (2.49)	1.67 (0.52)	14.18 (2.13)
	Motor group	8.58 (2.75)	11.12 (2.50)	2.12 (0.52)	3.04 (2.07)
	p-value	0.81	0.35	0.55	**0.002**
	95%CI	(-6.94 -8.77)	(-11.09-4.14)	(-2.01 -1.11)	(4.64-15.64)
	Effect size (G_{Hedges})	0.11	-0.49	-0.31	1.82

Somatosensory function

		**Em-NSA** /40	**PTT** /10mA	**TDT**-AUC	**WPST** total error degrees	**WPST** mean error degrees	**fTORT /42**
**T2-T1**	Sensorimotor group	1.12 (2.28)	-1.16 (0.89)	21.80 (8.16)	-93 (45)	-3.27 (2.17)	0.66 (1.97)
	Motor group	4.84 (2.40)	0.03 (0.98)	7.33 (8.94)	-92 (52)	-4.80 (2.31)	3.51 (2.17)
	p-value	0.29	0.41	0.26	0.99	0.64	0.35
	95%CI	(-10.96-3.53)	(-3.93-1.68)	(-11.39-40.34)	(-149-148)	(-5.29-8.36)	(-9.09-3.39)
	Effect size (G_{Hedges})	-0.55	-0.4	0.54	-0.01	0.23	-0.44
**T3-T2**	Sensorimotor group	1.63 (1.58)	-0.82 (0.63)	3.20 (5.18)	11 (31)	1.03 (1.56)	1.05 (1.34)
	Motor group	2.09 (1.79)	-0.73 (0.71)	-1.23 (5.93)	3 (34)	-0.03 (1.66)	1.06 (1.43)
	p-value	0.86	0.93	0.60	0.87	0.66	1.00
	95%CI	(-5.71 -4.80)	(-2.15-1.98)	(-12.90-21.74)	(-95-112)	(-3.92-6.04)	(-4.24-4.22)
	Effect size (G_{Hedges})	-0.09	-0.04	0.27	0.08	0.23	-0.002
**T3-T1**	Sensorimotor group	2.29 (2.32)	-1.65 (0.65)	23.23 (9.14)	-50 (53)	-3.27 (2.17)	1.75 (2.05)
	Motor group	6.59 (2.34)	-1.91 (0.78)	10.16 (10.30)	-96 (57)	-4.80 (2.31)	4.51 (2.17)
	p-value	0.22	0.80	0.36	0.57	0.64	0.37
	95%CI	(-11.43-2.84)	(-1.92-2.44)	(-16.29-42.43)	(-119-211)	(-5.29-8.36)	(-9.10 -3.58)
	Effect size (G_{Hedges})	-0.65	0.12	0.45	0.29	0.23	-0.42

Estimated mean and standard error of changes scores (T2-T1, T3-T2, T3-T1) are presented for both groups; p-values based on mixed models with age stroke onset (years) as covariate to evaluate differences between the change scores of both groups. Correction for multiple comparison was set on p<0,02. ARAT: action research arm test, FMA-UE: Fugl- Meyer assessment upper extremity section, SULCS: stroke upper limb capacity scale, Em-NSA: Erasmus modification of Nottingham sensory assessment, PTT: perceptual threshold of touch, TDT: texture discrimination test, AUC: area under curve, WPST: wrist position sense test, fTORT: functional tactile object recognition test.
