# Supplementary material for: Sensorimotor vs. Motor Upper Limb Therapy for Patients With Motor and Somatosensory Deficits: A Randomized Controlled Trial in the Early Rehabilitation Phase After Stroke
Source: Front Neurol. 2020 Dec 4;11:597666. doi: 10.3389/fneur.2020.597666 (PMC7746814; doi:10.3389/fneur.2020.597666)
Supplement: Supplementary Table 3 — Number of participants. Overview of number of participants included for each analysis. [file Table_3.pdf]

Supplementary table 3. number of participants included in analysis

	**ARAT** /57			**FMA** /66			**SULCS** /10			**ABILDHAND** (logits)			**fTORT** /42		
	**T2-T1**	**T3-T2**	**T3-T1**	**T2-T1**	**T3-T2**	**T3-T1**	**T2-T1**	**T3-T2**	**T3-T1**	**T2-T1**	**T3-T2**	**T3-T1**	**T2-T1**	**T3-T2**	**T3-T1**
Sensorimotor therapy (total)	19	18	19	17	16	16	16	16	15	16	17	15	19	18	18
Mild to moderate motor	10	9	10	10	9	9	9	9	8	9	9	8	10	9	9
Severe motor	9	9	9	7	7	7	7	7	7	7	8	7	9	9	9
Mild to moderate TDT	8	8	8	8	8	8	7	8	7	7	8	7	8	8	8
Severe TDT	11	10	11	9	8	8	9	8	8	9	9	8	11	10	10
Motor therapy (total)	17	16	16	15	15	15	16	15	15	17	16	16	17	16	16
Mild to moderate motor	8	7	7	7	7	7	8	7	7	8	7	7	8	7	7
Severe motor	9	9	9	8	8	8	8	8	8	9	9	9	9	9	9
Mild to moderate TDT	8	7	7	7	7	7	8	7	7	8	7	7	8	7	7
Severe TDT	9	9	9	8	8	8	8	8	8	9	9	9	9	9	9

	**Em-NSA** /40			**PTT** /10mA			**TDT**-AUC			**WPST** total error degrees			**WPST** mean error degrees		
	**T2-T1**	**T3-T2**	**T3-T1**	**T2-T1**	**T3-T2**	**T3-T1**	**T2-T1**	**T3-T2**	**T3-T1**	**T2-T1**	**T3-T2**	**T3-T1**	**T2-T1**	**T3-T2**	**T3-T1**
Sensorimotor therapy	16	18	15	19	18	19	19	17	17	19	17	17	17	17	17
Mild to moderate motor	8	9	7	10	9	10	10	9	9	11	9	9	9	9	9
Severe motor	8	9	8	9	9	9	9	8	8	8	8	8	8	8	8
Mild to moderate TDT	7	8	7	8	8	8	8	7	7	8	8	8	8	8	8
Severe TDT	9	10	8	11	10	11	11	10	10	11	9	9	9	9	9
Motor therapy	16	15	15	17	15	15	17	15	15	16	15	15	15	15	15
Mild to moderate motor	8	7	7	8	6	6	8	6	6	8	7	7	7	7	7
Severe motor	8	8	8	9	9	9	9	9	9	8	8	8	8	8	8
Mild to moderate TDT	8	7	7	8	7	7	8	7	7	8	7	7	7	7	7
Severe TDT	8	8	8	9	8	8	9	8	8	8	8	8	8	8	8

Number of participants for total group and subgroup analysis. Subgroup analysis is represented for baseline mild to moderate or severe motor impairments and baseline mild to moderate or severe texture discrimination impairments; ARAT: action research arm test, FMA-UE: Fugl- Meyer assessment upper extremity section, SULCS: stroke upper limb capacity scale, Em-NSA: Erasmus modification of Nottingham sensory assessment, PTT: perceptual threshold of touch, TDT: texture discrimination test, AUC: area under curve, WPST: wrist position sense test, fTORT: functional tactile object recognition test.
